# The Pulse

**Published:** 1871-02

**Authors:** 


The Pulse.—The pulse was first noticed by Galen. It is slower in the inhabitant of the country than in one of the city. In the West Indies it is 100; in Greenland as slow as 40; and is slowest in the cold climates. It is slower in the winter than in summer. Is slow in the morning, quick at noon, and more frequent at night.—*Medical and Surgical Reporter.*

